# Visually Significant Dimensions and Parameters for Gloss

**DOI:** 10.3390/jimaging10010010

**Published:** 2023-12-29

**Authors:** Donatela Šarić, Aditya Suneel Sole

**Affiliations:** 1 Prepress Department, Fogra Research Institute for Media Technologies, 85609 Aschheim, Germany; 2 Department of Computer Science, Norwegian University of Science and Technology, 2815 Gjovik, Norway; aditya.sole@ntnu.no

**Keywords:** gloss, appearance, gloss perception, gloss dimensionality

## Abstract

The appearance of a surface depends on four main appearance attributes, namely color, gloss, texture, and translucency. Gloss is an important attribute that people use to understand surface appearance, right after color. In the past decades, extensive research has been conducted in the field of gloss and gloss perception, with different aims to understand the complex nature of gloss appearance. This paper reviews the research conducted on the topic of gloss and gloss perception and discusses the results and potential future research on gloss and gloss perception. Our primary focus in this review is on research in the field of gloss and the setup of associated psychophysical experiments. However, due to the industrial and application-oriented nature of this review, the primary focus is the gloss of dielectric materials, a critical aspect in various industries. This review not only summarizes the existing research but also highlights potential avenues for future research in the pursuit of a more comprehensive understanding of gloss perception.

## 1. Introduction

Visual appearance is the result of the interaction between the physical properties of an object, the illuminating characteristics of the light, and the human visual system. The main appearance attributes are considered color, gloss, texture, and translucency [1,2]. Visual appearance plays a crucial role in our perception of the world around us. The appearance of an object provides us with information about its identity and function but also influences our emotional and behavioral responses toward the observed object. The study of the visual appearance of objects has been a central topic in psychology, neuroscience, computer vision, design, and more. When light strikes a surface and reflects, it can result in different types of reflection, depending on the surface properties. A perfectly polished mirror reflects light uniformly at the same angle in the opposite direction, called the specular direction. High-gloss surfaces also produce concentrated, coherent reflection in the specular direction, and it creates highlights on glossy surfaces. In contrast, rough or irregular surfaces cause diffuse reflection, scattering incident light in various directions. Unlike the focused reflections of specular and mirror reflection, diffuse reflection results in soft, nondirectional light scattering. In most cases, the reflected light consists of diffuse and specular reflection. From the specular reflection, we obtain gloss information, and from the diffuse reflection, color information.

From that, it can be noted that the appearance of an object is a complex and multidimensional phenomenon that can be difficult to quantify objectively. However, the measurement of appearance is crucial for many industries. There are various methods and techniques for measuring appearance, ranging from subjective evaluation by human observers to objective measurements using advanced imaging and analytical techniques. The subjective evaluation of appearance involves the assessment of visual properties by human observers. This type of evaluation is typically performed using a visual inspection or rating system, where trained or untrained observers rate the appearance of objects on a scale or using descriptive terms. While subjective evaluation can provide valuable information about the perceived appearance of objects, it is highly dependent on individual perception and can be influenced by factors such as lighting, background, and other parts of the setup of the psychophysical experiment. On the other hand, objective measurements of appearance involve the use of advanced imaging and analytical techniques to quantify the visual properties of objects. These techniques can provide precise and standardized measurements of color, gloss, translucency, and texture, but a measurement of any appearance attribute needs to take the underlying dimensionality into consideration. Numerous studies have demonstrated that color perception can be effectively described using three dimensions. However, when it comes to describing gloss, the dimensionality remains uncertain and difficult to define [3,4,5].

Gloss is usually considered to be the second important appearance attribute, right after color [6]. It gives us information about the object’s properties, such as surface shape, solidity, moisture (wet or dry), roughness, position in the environment, etc. Additionally, gloss is a second-order attribute, which means we do not have receptors for gloss on our retina; instead, we get the color information of the surface, and the information is further reconstructed in our brain as a mat or glossy surface. According to ISO 2813, gloss is an *“optical property of a surface, characterized by its ability to reflect light specularly”* [7]. The CIE (Commission Internationale de l’Eclairage) definition of gloss is that gloss is a “mode of appearance by which reflected highlights of objects are perceived as superimposed on the surface due to the directionally selective properties of the surface.” [8]. With this CIE definition from 1987, gloss was no longer considered a purely physical property of a material; instead, the CIE defined gloss as a perception that can be quantified and associated with the geometrical properties of a surface. In the work from Chadwick and Kentridge, a comprehensive exploration of the factors and mechanisms contributing to gloss perception is described [9]. Building upon Chadwick and Kentridge’s research, this literature review paper extends their work by focusing more on the experimental setup and methodologies that were used in the reviewed work. By examining the setup of the experiment, we aim to provide a complementary perspective for further enhancing the study of gloss perception.

## 2. Gloss Measurements

**Figure 1:**
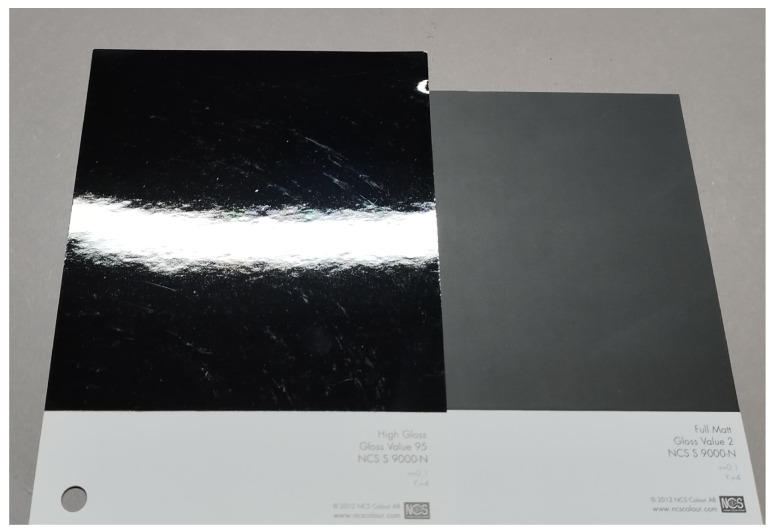
Samples with the same color and different specular gloss (95 GU on the left and 6 GU on the right) measured at a 60° incidence angle.

The concept of gloss has long captivated researchers and professionals in various fields, including material science, surface engineering, graphic design, etc. Over the years, various methods have been developed to measure and quantify gloss, providing valuable insights into the physical and perceptual aspects of gloss. In this chapter, we explore the different approaches to gloss measurement, divided into two categories: physical gloss measurements and perceptual gloss measurements. By examining these two perspectives, we can gain a comprehensive understanding of gloss and its multifaceted nature. In Figure 1 two samples with the same color and different glossiness are shown.

### 2.1. Physical Gloss Measurements

For decades, optical instruments have been utilized by various industries to assess different properties of surface gloss, and they continue to be employed to this day. This demonstrates their effectiveness in tasks such as quality control and the detection of defects or anomalies in production processes. Specular glossmeters are optical instruments that are used to measure the specular gloss of a surface. Specular gloss refers to the amount of light that is reflected from a surface in a mirror-like manner, as opposed to diffuse reflection, which scatters light in many directions. Specular gloss is a ratio of the luminous flux reflected from the specimen to that reflected by a glass surface with a refractive index of 1.567 at a wavelength of 587.6 nm in the specular direction and is indicated as Gloss Unit (GU) [7].

$$\mathrm{Gloss}=\frac{R_{\mathrm{sample}}}{R_{\mathrm{standard}}}\times 100$$

Different standards recommend different angles of measurement, reflecting different use cases and demonstrating the complexity of the problem. The American Society for Testing and Materials (ASTM) has played a significant role in the development of gloss measurement. Their technical work has led to the development of a widely adopted standard, also known as the ASTM Method [7,10]. However, it has been observed that there is a lack of correlation between the visually assessed gloss data and the measured gloss data across the entire range [11]. The complexity of gloss lies in the relation between the physical stimulus and perceived gloss, which is complex and still not fully understood [12]. While specular glossmeters provide a single-value measurement (one dimension) of the specular gloss of a surface, these instruments have certain limitations that make them inapplicable for the soft metrology of surface gloss. Soft metrology encompasses measurement techniques and models designed to quantitatively assess properties influenced by human perception, involving any of the five senses (sight, smell, sound, taste, and touch) [13]. For instance, the limited dynamic range of glossmeters compromises their ability to accurately characterize surfaces, especially for very mat samples, where the read-out is dominated by diffusely reflected light [14,15]. Thus, gloss measurements with one-dimensional glossmeters are suitable for measuring gloss differences between samples, rather than for providing an absolute scale for gloss. However, it is worth considering the possibility of mathematically transforming these measured gloss values into a perceptual uniform gloss scale to enable an absolute glossiness comparison. However, for more complex surfaces and luxurious objects where there is a need for more extensive research, it becomes necessary to consider multiple dimensions. In such cases, the Bidirectional Reflectance Distribution Function (BRDF) is used in order to capture the comprehensive characterization of their gloss [16].

#### 2.1.1. BRDF

The BRDF, or Bidirectional Reflectance Distribution Function, is a mathematical function used to describe how light is reflected from a surface in different directions [17]. The BRDF describes the ratio of the light reflected from a surface in a particular direction to the light incident on the surface in a particular direction. In other words, it describes how the surface reflects light in different directions, considering the angle of incidence and the angle of reflection. The BRDF is a function of four variables: the incident angle, the viewing angle, the wavelength of the light, and the polarization of the light [18,19,20]. BRDF measurements in general provide a complete angular distribution of how light is reflected from the surface in all directions. This includes both the specular reflection and the diffuse reflection components. BRDF measurements allow for a more thorough understanding of how light interacts with a surface and can be used to model the surface’s appearance under a wider range of lighting conditions. BRDF measurements with defined subsets, i.e., angle combinations (deduced from different use cases), are commonly used in research and development for material characterization, as well as in industries such as aerospace, automotive, and semiconductor manufacturing. In these industries, BRDF measurements can be used to optimize surface treatments and coatings, ensure uniformity and consistency in surface properties, and improve the accuracy of simulations used for product design and testing. However, BRDF measurements are typically more complex and time- and money-consuming than specular gloss measurements [14,17,21,22]. From the BRDF, important parameters can be computed, like color, specular reflection, specular peak, distinctness-of-image gloss, image clarity, specular peak, luminous flux, haze, and some other parameters that are not part of any standard (instead, companies develop their own parameters for appearance description).

**Figure 2:**
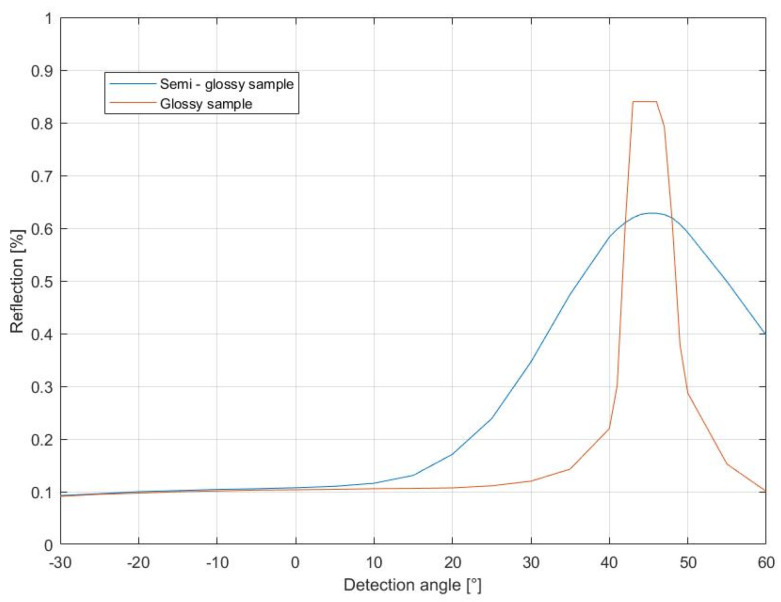
Example of BRDF measurements of two 2.5D printed color patches with the same color and different glossiness. The BRDF measurements were taken at a 45° incidence angle and 580 nm wavelength.

In Figure 2, two BRDF measurements are shown. The measured samples are mat and glossy 2.5D printed samples. The samples were measured with the incidence angle at 45° at the wavelength of 580 nm. For easier interpretation of the reflection distributions, the reflection scale is logarithmic. From the height of the specular peak, we can notice which BRDF curve represents which object. The specular peak is the most important component of gloss. For different materials, the specular peak is strongly dependent on the refractive index (n). The higher the refractive index, the higher the specular peak. The size of the peak from a measured surface also depends on the angle of incidence. The peak increases when the incidence angle increases [6]. The specular reflection will be bigger when illuminating the object under a grazing angle (e.g., 85°). Therefore, ISO 2813 standard recommends three incidence angles, 20°, 60°, and 85° geometries [7]. The 60° geometry is used for intercomparing the gloss of most specimens. The 20° geometry is advantageous for comparing specimens that have a 60° gloss higher than 70 GUs (Gloss Units), whereas the 85° geometry is used for comparing specimens for sheen or near-grazing shininess. It is mostly applied when the 60° geometry gloss is lower than 10 GUs.

In a work from 1998, Dana investigated the complex nature of surface reflectance and texture and proposed a comprehensive framework for understanding and modeling the properties of real-world samples and the dependence of appearance on the geometry of imaging conditions [23]. In the work, they developed a simple system sustainable for simultaneous BRDF and BTF measurements. The texture representation, called BTF (Bidirectional Texture Function), is discussed. In the work, an object was measured from different illumination and detection angles. The image was captured with a video camera with a frame grabber. The pixel values are converted to radiance values using a calibration scheme. Further, the calibrated images served as the BTF measurements, and the images were averaged over the sample area to obtain the BRDF. For detection, a CCD camera is used.

In comparison with specular glossmeters, BRDF measurements and their setup are complex, and a lot of parameters can influence the results. In 2015, Obein et al. investigated measurements of two high-level goniospectrophotometers [24]. The BRDF values were measured for mat and high-gloss samples independently on the two devices. The results show differences in the specular peak, even for the mat sample. For the high-gloss samples, the differences are more expressive. A larger illumination spot and detection aperture of one of the goniospectrophotometers results in an angular broadening and lower peak value. Furthermore, by rendering the results into luminance maps, the visual differences were more expressed in the high-gloss samples.

While there are some standards related to the BRDF measurements that provide guidelines and procedures for measuring the BRDF [25,26,27], sometimes the impact of roughness and anisotropy is not addressed. Additionally, there may be some disagreement among researchers regarding the appropriate mathematical models or parameters to use when analyzing BRDF data [28,29]. It is important to be aware of these limitations and take appropriate steps to mitigate their impact on BRDF measurements. This may include selecting appropriate measurement equipment, following best practices for sample preparation, controlling environmental conditions, and ensuring appropriate operator training and qualifications [30].

Today, the capture of appearance has improved, but BRDF measurements are still time- and money-consuming. Furthermore, the calculation of gloss values (and other values) from BRDF measurements is still challenging. For quick capture, Dana set up a capture system with a mirror-based camera as a detector. For the illumination, a projector with one light source was used. In between, a parabolic mirror was placed. The specialty is that the mirror cameras can have different geometry, for example, the texture camera which uses a concave off-axis parabolic mirror to replace the angular movements required in a gonioreflectometer. Another mirror-based camera is a multiview radial imaging system that obtains a dense sampling of viewing directions using a conical curved mirror placed in the light path of the camera. The third capture setup is based on multiple cameras and multiple light sources where the turntable rotates, or another setup where there are no moving parts [31]. This type of measurement allows for a more comprehensive characterization of how the reflectance properties of a surface change across its spatial extent. By understanding the spatial variation in BRDF, one can gain a deeper understanding of how gloss properties may vary across a surface, providing valuable information for various applications such as material design, surface inspection, and computer graphics rendering.

In 2021, Saha et al. developed a goniospectrophotometer for BRDF measurements on a microscopic scale (μBRDF) [32]. The device uses a Laser-Driven Light Source (LDLS) as a light source and a Konica Minolta CS-2000 spectroradiometer as a detector. The sample is placed on a six-axis robot arm. When the light gets reflected from the sample, it goes through a custom optical system, which enables a field of view of 300 μm when using the 0.1° CS2000 field of view setting. The diameter of the area measured by the CS-2000 is 263 μm ± 5 μm. With the development of softproofing in different industries, the μBRDF enables more accurate fiber and hair characterization of all appearance attributes, together with gloss.

#### 2.1.2. Perceptual Gloss Measurements

From the previous chapter, it can be noted that quantifying an object’s appearance is a challenging task. While objective measurements such as BRDF and specular glossmeters provide valuable information about the physical properties of surfaces, they do not directly capture the perceptual experience of gloss. Gloss perception involves not only the physical properties of the surface but also the cognitive and psychological processes of the observer. Psychophysical measurements bridge the gap by incorporating human perception and subjective judgments. It is unlikely that one physical scale for “visual appearance” will be possible, and it is necessary to find physical parameters that correlate with the four main appearance attributes, and most importantly, that the physical attributes can be measured [33]. Nonetheless, researchers continue to explore and refine models that provide valuable insights into the correlation between physical properties and human perception of the four main appearance attributes.To ensure that the stimuli used in psychophysical experiments are controlled and consistent, researchers often use specialized setups and equipment to present and measure the stimuli. These setups can vary depending on the type of experiment being conducted.

In addition to equipment, the setup of a psychophysical experiment can also include the design of the experimental task and the selection of the participant sample. The experimental task should be designed to elicit consistent and meaningful perceptual responses [34]. The setup of a psychophysical experiment is critical to ensuring that the stimuli used are controlled and consistent and that the perceptual responses obtained are meaningful and accurate. By carefully designing and implementing a setup for a psychophysical experiment, researchers can gain valuable insights into the relationship between physical stimuli and perception. Unfortunately, psychophysical experiments for appearance, including gloss judgment, still lack standardization and research. However, standards such as ASTM D4449 offer a valuable contribution to the field [35]. ASTM D4449 provides a standardized method for the visual evaluation of gloss differences between surfaces of similar appearance. The standard provides guidelines for simple tasks, but it may overlook other factors that can influence gloss perception, such as surface texture, lighting angles, or environmental conditions. As a result, the standard’s ability to address the full range of gloss perception may be limited. Furthermore, the ASTM D4449 standard does not provide detailed guidelines or procedures for controlling and standardizing experimental conditions, such as illumination or observer training. Inconsistencies in these factors can affect the reliability and reproducibility of gloss evaluations, potentially leading to variations in results across different experiments or laboratories. The lack of explicit guidance in these areas leaves room for differences in implementation and introduces potential sources of error or bias. The present review will show different setups for the psychophysical evaluation of gloss and their outcomes based on the literature references on the topic [36].

Some work that has been conducted in visual gloss evaluation shows that the gloss judgment by the observers does not change with the change in the illumination angle or the angle of observation. Billmeyer and O’Donnell were the first to point this out [37]. In 2004, Obein et al. conducted an experiment using the maximum likelihood difference scaling (MLDS) procedure [12]. The authors chose this scaling method because it showed successful results in quantifying color differences in tristimulus space and the perceived distortion of an image as a function of compression [38,39]. The aim of the experiments was to test the sensitivity of the visual system to the parameters of the specimens’ surfaces. Therefore, the question in the psychophysical experiment is not related to gloss; instead, the observers were asked *which of the two pairs exhibits a larger difference.* The experiments were performed with 20° and 60° illumination angles to match the standards for specular gloss measurements [7]. The results show that the observers’ gloss sensitivity is lower in the semimat and semigloss ranges (the authors also call it the intermediate range) and that the sensitivity increases with the gloss of the sample. The results suggest that observers obtain information about gloss and appearance other than luminous flux, which is reflected from the observing surface, and a single scale is not sufficient to describe the obtained results from the experiments. However, the results show that there is no significant difference between the results obtained in the 60° and 20° configurations. These results provide evidence for “gloss constancy”. Some other work was carried out that supports this theory, like the work from Nishida and Shinya in 1998 [40] and Fleming at al. in 2003 [41], only with the difference that Obein et al. used real surfaces.

The work can also be a notable example of how the setup of the experiment can affect the results. For example, Obein’s psychophysical gloss scale matches the scaling from other authors’ work, like Judd and Hunter’s [42] for the mat samples and Harrison and Poulter’s in the intermediate part [43], but it does not match with Billmeyer and O’Donnell’s [37]. All the mentioned studies use real-world specimens in their experiments. The used samples are paper-like, painted plaquettes, paint panels, and glass. Also, the difference is in the experimental setup. Obein used MLDS with pair-to-pair comparison, Billmeyer, O’Donnel, and Leloup magnitude estimation, and the Hunter ranking method.

It is already noted that different setups can produce equivalent results, but they are not 100% the same. In the realm of psychophysical experiments for gloss observation, it is important to acknowledge that computer simulations of object gloss may introduce certain deviations from real-world scenarios. One notable aspect to consider is the potential tonal compression that can occur during the rendering process. Tonal compression can lead to differences in the visual appearance of simulated objects compared with their real-world counterparts. Moreover, the specific 3D rendering model applied can significantly impact the perceived glossiness of objects within the simulation. Besides that, studies show that there is indeed a relationship between perceptual attributes and visual, tactile, and subjective attributes [44].

Research conducted by Ged et al. [45] showed that the gloss constancy can be broken for mat objects when the illumination is diffuse. When illuminating a mat object with a diffuse light source, the light comes from more directions. This causes a decrease in the perceived gloss of mat and semimat objects. We can conclude that the incoming light and the nature of the light play a significant role when evaluating the gloss. Ged et al. explained this in their work [46]. He compared two setups for psychophysical experiments. Two equivalent sets of coated paper were used in the experiment in two different institutions. Both institutions performed the experiment with diffuse and specular light sources. The paired comparison was used as the evaluation method: at one institution, the maximum likelihood difference scaling, and at another one, the paired comparison described by Scheffe in 1952 [38,47]. The results show that, despite the differences in illumination ratio between the specular and diffuse ambient scales, the psychometric scales at the second institute in the experiment overlap. It is noted that the presence of a virtual image of light sources on the sample greatly affects the gloss appraisal.

The human eye is used to perceive glossiness under natural light and conditions. Van Assen et al. tested how observers perceive the appearance of round objects (spheres) with illumination with artificial properties [48]. The rendered objects were illuminated with different illumination geometries, which can be like the ones that can be found in real life, but also some artificial, like circles, dots, rings, squares, and a window. They concluded that when illuminating objects with unnatural illumination, the observers lose their sensitivity to gloss perception. This proves that the gloss is implemented in our cognitive system, and by illuminating objects with artificial illumination, the cognitive system has problems reconstructing the scene [49,50]. Later, in 2019, Faul proved that the illumination type has a strong influence on the perceived glossiness. Results from Faul’s work suggest that Fresnel-BRDF is a better approximation than the Ward model, especially when using homogeneous illumination [51]. Furthermore, the author implies that the glossiness of metallic and dielectric materials differ fundamentally with respect to Fresnel effects and that, when judging the glossiness of metallic and dielectric materials, we use different cues and mechanisms in both material classes. Unfortunately, for the three psychophysical experiments, the exact experiment question is not stated.

Since a change in diffuseness can result in a difference in the appearance of an object, it is important to determine the diffuseness condition that is most suitable for reproducing the surface appearance of an object. In 2022, Mizushima and Mizomaki investigated which light diffuseness faithfully reproduces the surface appearance of an object as seen in a natural environment [52]. Five observers evaluated the fidelity and “ideality” of the object’s appearance under four or five diffuseness conditions. The objects were made from different materials, namely polyresin balls, fur charms, wooden cubes, and stainless-steel cubes. In their work, the most faithful reproduction was under moderate diffuseness; furthermore, the ideal diffuseness depends on the material of the object. This implies that the choice of materials for the psychophysical experiments can be crucial when evaluating the appearance. In Table 1, an overview of the mentioned papers, together with the details of the psychophysical experiments, is shown.

The papers mentioned in this chapter focus on the influence of different setups for gloss evaluation. The samples that have been used for the psychophysical evaluation vary in material type, but it can be noted that the illumination type and illumination geometry strongly influence the gloss perception, contrary to the angle of illumination, which seems to be irrelevant for gloss assessment due to the gloss constancy. It is suggested that when performing a psychophysical experiment, the setup of the experiment should resemble as closely as possible the conditions in which the observers naturally perceive objects, since we are used to perceiving gloss in nature [48]. Besides illumination art, the scene setup of the experiment seems to have a big impact on the results. Ged et al. suggest that when evaluating the gloss, a mesh should be used, and the reflection of the light source should be visible in the samples [46].

## 3. Gloss Dimensionality

The measurement of gloss is a crucial aspect in many industries, allowing objective and precise quantification of visual properties. While subjective evaluation by human observers can provide valuable information about the perceived appearance of objects, objective measurements using advanced imaging and analytical techniques offer greater precision. The most commonly used measurement device in the industry for gloss measurement is the specular glossmeter, which measures the specular gloss of a surface by comparing the reflected flux from the measured surface to the reflected flux from the reference standard in the same geometry. These measurement devices make it possible to differentiate between surfaces of similar appearances. However, these instruments possess limitations that make them unstable for the soft metrology approach [6,14]. Understanding the dimensionality of gloss is important for various applications, including material design, object recognition, and image rendering to create realistic surfaces that mimic real-world materials [53].

In 1930, Pfund first pointed out that the perception of gloss may be defined as multidimensional [54]. He noted that the gloss is related to the contrast between the specular reflection and the lightness of the surrounding surface area. After that, Hunter pointed out that not only two but at least six visual criteria contribute to the final gloss appearance, namely specular gloss, contrast gloss, sheen, absence-of-bloom gloss, distinctness-of-image (DOI), and surface uniformity [6]. Hunter’s work greatly influenced the design of gloss measuring devices in industries, but there is a common mistake of confusing Hunter’s description of gloss with the factors that make it look like that. In other words, Hunter talked about how gloss looks and not what makes the gloss look that way [12].

In 2001, Ferwerda et al. [55] extracted DOI and contrast gloss as crucial parameters for gloss perception. Contrast gloss is, according to Hunter, “the perceived relative brightness of specularly and diffusely reflected areas”. The multidimensional scaling (MDS) showed two dimensions for gloss perception. Namely, one dimension was correlated with the distinctness-of-image (DOI) and the other with contrast gloss. The results indicate that, when observing gloss, observers do not perceive only the specular reflection; instead, the surroundings of the specular reflection affect the final gloss perception too. Therefore, darker (black) objects with the same intensity of specular reflection are perceived as glossier than lighter (white) objects. Furthermore, by observing the gloss, we are observing the light and image reflection from the object. The sharper the image reflection, the glossier the object will be perceived. Therefore, ASTM recommends using a directed light source with a mesh installed under the light source, so observers have a pattern and hence defined contrasts as reference [35]. In 2010, Ged et al. investigated if gloss properties can be exploited as an appearance attribute to identify and discriminate between real materials [56]. They designed a psychophysical experiment to determine which part(s) of the BRDF humans observe when observing materials. The samples that were used in the psychophysical experiment were opaque Plexiglas samples (PMMA) with several types of roughness on the surface. It was shown that using only one parameter is not enough to explain the perceived visual judgment of appearance. Furthermore, results from the multidimensional analysis show that gloss is indeed multidimensional, and the human visual system can select different components within gloss perception. They propose that the three principal components of gloss perception are luminous flux, reflection haze, and the microfacet distribution of the surface.

Leloup et al. investigated the cues for gloss perception [57]. For the experiment, virtual stimuli were used based on measurements of the real-life samples. The stimuli were created based on the measurements of flat glass and papers with different glossiness. In total, 16 samples were created by applying different illuminations to the four samples. Using paired comparison, a gloss scale was made. From the gloss scale, it can be inferred that gloss perception is impacted by variations in distinctness-of-image and luminance (Y). When only one of the two cues was different, gloss differences were reported by the observers. The Principal Component Analysis (PCA) revealed duality among observers. One group of observers largely utilized the luminance cue, while the other group primarily used the DOI cue to assess surface gloss.

In 2020, Toscani et al. investigated the dimensionality of gloss by creating different virtual stimuli [58]. The results from two psychophysical experiments show that the observers’ responses correlate with three dimensions, namely specular gloss reflection, lightness, and metallicity of the sample. This could be expected since the stimuli were created by using the ABC model [59]. Crucially, these three dimensions were characterized by different physical properties that shape the reflection curve (BRDF), as indicated by the parameters of the ABC model. The ABC model proposed by Löw et al. is based on the Beckmann distribution function, which is commonly used to describe the roughness of a surface. The Beckmann function is used to calculate the surface microfacets’ orientation, which determines the surface’s reflection behavior [60]. The proposed model consists of three components, which are referred to as the ABC model for rendering. The first component is attenuation, it describes how light is absorbed as it travels through the surface and is affected by the surface’s thickness and transparency. The second component uses the Beckmann distribution function to determine the orientation of the surface microfacets, which affects the surface’s reflectivity. The third component is clarity, which describes how the surface reflects light and is affected by gloss, roughness, and viewing angle. The authors do not report conducting any psychophysical experiments.

Table 2 includes a summary of the papers reviewed in the Section 3. Depending on the viewing task, one, two, or three dimensions are important to explain the pertinent experiment design. In the industry, for gloss control, most of the time, the one-dimensional gloss measurements are enough. On the other hand, for more luxurious and complex products (e.g., automotive industry), more complex methods are sometimes used to better describe and understand the appearance of more complex surfaces. In the papers mentioned in this paragraph, in almost all experiments, virtual stimuli were used, namely 3D objects (spheres or bumpy objects). An exception is one work where real-world materials were used (PMMA) [56]. In both cases, the gloss was described as multidimensional. While in the first two works, pair comparison was used as a psychophysical scaling method, in the last-mentioned work, observers had a matching and rating task for gloss evaluation. Furthermore, one of the papers differs in the question that was asked to the observers. Namely, the authors did not ask the observers about the gloss difference; instead, the question pertained to the whole surface appearance of the used samples. The author’s aim was to better connect gloss and its correlation with other appearance attributes.

## 4. Surface Appearance Characteristics

In the pioneering work of R.S. Hunter, he introduced a set of systematic cues to describe and quantify gloss appearance. These cues serve as a foundation for understanding the visual perception of gloss. Namely, he introduced specular gloss, sheen, contrast gloss or luster, absence of bloom, and distinctness of image (DOI) [6]. These attributes further contribute to the comprehensive understanding of gloss perception and its impact on visual interpretation.

### 4.1. Haze Gloss

Haze gloss has gained considerable attention in numerous studies due to its strong impact on visual perception. It can be defined in two ways: one is the reflectance haze, and the second is the transmission haze. In this section, due to the topic of this work, only the reflection haze is taken into consideration. Reflection haze is the scattering of light on a glossy surface (e.g., plastics, metallic surfaces, etc.) responsible for the apparent reduction in contrast of viewed objects [61]. According to the ASTM standard, haze is “*for a specified specular angle, the ratio of flux reflected at a specified angle (or angles) from the specular direction to the flux similarly reflected at the specular angle by a specified gloss standard*” [62]. In the ISO Standards, the reflection haze is *a milky opalescence in high-gloss or clear coatings* [63].

Hazy gloss reflection appears as a halo surrounding the core of specular reflection on high gloss samples. Psychophysical experiments performed on this part of gloss appearance prove that haziness is more complex than the haze measurements retained by ASTM, as the hazy gloss is not directly dependent on physical parameters [64,65]. In materials science, haze gloss is often used to describe the appearance of plastic films or coatings that are used in a range of applications, such as packaging, automotive, and electronics. These materials often need to have a balance between gloss and haze properties to provide the desired visual appearance and functional performance. Therefore, this aspect of gloss has gained a lot of interest from researchers.

In 2022, González-Leal et al. proposed a novel method for visual haze evaluation for stainless steel [66]. The system consists of a high-contrast Ronchi-type pattern, with its normal forming an angle with respect to the normal of the surface. A camera is placed on the other side with the optical axis aligned with the specular reflection of the pattern. The system is a noncontact measurement system, and it is based on image analysis. Namely, the reflection of the pattern from the specimen is evaluated. The authors provide results from psychophysical observations, but only the authors were the observers. The authors provide sufficient detail for readers to replicate their methods and findings. The study is, however, limited to a specific type of steel, so the applicability of the proposed method to other materials is not fully explored.

In 2017, Vangorp et al. suggested that haze perception is a disjunction of the specular reflection of the BRDF into the specular core and the surrounding halo [67]. The study is based on the idea that hazy gloss occurs when a glossy surface scatters light in a diffuse manner, resulting in a foggy or misty appearance. In the work, they conducted three psychophysical experiments, first to match one material to the other in terms of the sharpness or blurriness of the reflections and then to select the object that looks different from the other two in terms of the sharpness and blurriness of the reflection. The third part of the experiment was to rate the presented material on six different continuous scales related to gloss appearance (glossy versus mat, sharp versus blurry, not hazy versus hazy, polished versus unpolished, low versus high friction, coated versus not coated). It can be noted that the authors do not use the word haze when conducting the psychophysical experiment. Instead, with these simplified questions, the authors focus the question on the haze parameters and not only on the haze. The results from the discrimination and rating tasks support the idea that observers are sensitive to the additional complexity of two-component BRDFs, especially when there is a big contrast in sharpness between the specular core and the surrounding halo (also called bloom). Therefore, Barla et al., using these results, presented a composite BRDF model for the rendering of hazy gloss [68].

The authors employ a specular BRDF based on two Ward BRDF components, one for narrow widths and the other for wide widths, and most importantly, the model is based on psychophysical parameters of haze appearance. The haze effect is controlled independently of the specular reflection (BRDF peak), granting control over spatial variations in haze. The model affects the global reflectivity; hence, it also indirectly modifies the refractive index of the used material. The approach is simple since it uses already existing models. By using a pair of the Cook–Torrance components [69], the fitting error was reduced. However, the only valid physical interpretation of their model is that of a mixture of distributions, such as partially polished single-layered materials. Due to the Fresnel term, the model does not work on more-layered materials. Furthermore, the authors of the work claim that different layers can affect perceived haziness, which makes the perception of haze more complex. In both works reviewed in this chapter, the authors provide a thorough explanation of the theoretical basis for their study, as well as clear descriptions of their methods and results. The paper provides valuable insights into how hazy gloss affects our perception of visual stimuli and may be of interest to researchers in the fields of vision science, materials science, and product design.

In the table below, a short overview of papers mentioned in this chapter is shown (Table 3).

### 4.2. Distinctness of Image (DOI)

**Figure 3:**
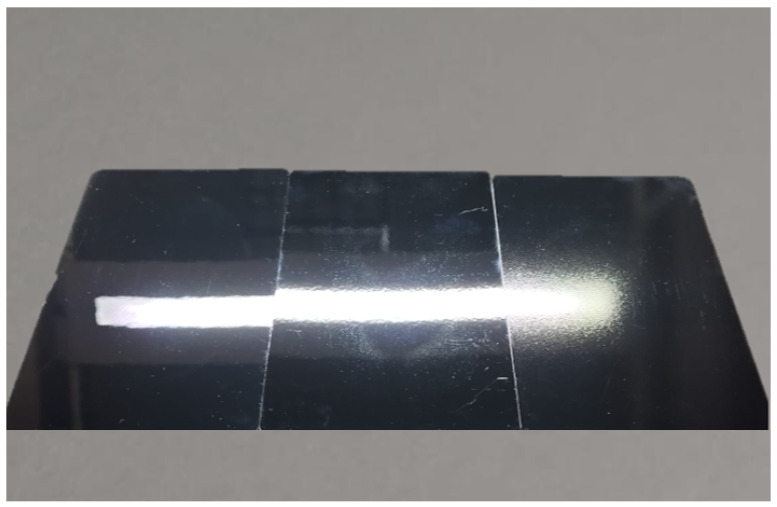
Samples with the same color and different distinctness-of-image gloss.

The distinctness of image is a fundamental concept in the realm of gloss perception, extensively investigated in various studies for its profound influence on the perceived sharpness and clarity of reflected images on surfaces. According to ISO 20791-3.2:2023, DOI is *"the degree of sharpness of an image reflected by a specimen or transmitted through a specimen”.* In some industrial fields, DOI is sometimes called image clarity [70]. A surface with high DOI will reflect the image of an object with high clarity and sharpness. (Figure 3) There are several setups for measuring DOI. The first one is goniometric, which is described in the ASTM D5767 standard [71]. A measurement device with a narrow aperture measures the slight light reflection of the specular angle (±0.3°). The DOI is measured by comparing the sharpness of the reflection of a standardized test pattern on the painted or coated surface with the sharpness of the pattern on a highly polished black glass reference standard. The second category of DOI measurement setup includes all the variations of the goniometric measurements, e.g., projecting the light through a narrow slit onto a specimen, and the reflected image is measured through a sliding filter to provide a value of image clarity. One example is the Canon Surface Reflectance Analyzer [72]. Besides this method, a pattern of light (usually parallel lines) can be projected onto the specimen and the reflected image evaluated. An example of this is the Rhopoint TAMS [73].

The last category of DOI measurement is by using an optical profilometer. This method includes scanning the sample with a narrow-beam light source (solid-state laser diode). The output of this method is an optical profile of the structure. From this profile, various DOI measures at different structure size scales are obtained using bandpass filtering. This method of measuring the DOI can be found in the BYK-Gardner Wave Scan [74].

Whichever setup is used for the DOI measurement, the results are influenced by a range of factors, including the composition and structure of the material, the surface texture, and the angle of incidence and reflection of the light. For example, a smooth and uniform surface will typically have a higher DOI value than a rough or irregular surface, as the smooth surface will reflect light more uniformly and create a clearer image. From the papers reviewed in some previous sections, it can be noted that distinctness-of-image gloss is an important parameter in gloss perception and also a dimension in gloss evaluation. In 2011, Lu et al. investigated the relationship between the distinctness of the image and the texture of the coating [75]. In the paper, the authors note that DOI is an important factor in the perceived quality of coated surfaces, but little is known about how the texture of the substrate affects DOI. They conducted experiments using steel substrate sheets with different surface textures and coated them with organic coatings of varying DOI. The results from the work show that the texture of the surface had a significant impact on the DOI, namely that the texture of the substrate sheets is inversely proportional to the coating DOI. They suggest that surface roughness could be used as a predictor of the DOI of coatings on that substrate. Overall, the paper provides evidence for a relationship between the DOI of organic coatings and the texture of the substrate sheet. Unfortunately, the results are based on measurements of roughness and DOI, and no visual observations are made to connect the results with the visual estimation of DOI and texture.

In 2005, Tse and Briggs [76] developed a new instrument for the distinctness of image measurement. The authors note that traditional metrics for image quality such as resolution, contrast, and color accuracy do not always correlate with the image quality perceived by humans. They propose the use of a DOI metric that measures the ability of human eyes to distinguish between two similar regions in an image. The method that their device uses is a DIAS (Distinctness of Image Analysis System) Method, where a sharp edge is projected onto the specimen’s surface and the reflected image is captured with a camera. The output of the measurement device is a reflectance profile obtained and analyzed to obtain a measure of the DOI. The performance of the instrument is compared with other methods for measuring the DOI and visual inspection, yet there is no statement that a psychophysical experiment is conducted, and it is unclear how the data for the visual assessment are obtained. While the paper is brief and the results are not presented in detail, the authors provide a clear and concise description of their setup and its potential applications.

Gruber and Buder-Strisznigg introduced a measurement method for the distinctness of images using the Intensity Profile Analysis (IPA) [77]. The system is based on a projection of a line chart onto the specimen. The line chart is printed on a transparent plastic sheet and illuminated from the back by a white LED array. The reflection from the specimen’s surface is captured with a CCD camera. From the intensity profiles, contrast profiles are calculated. The authors claim that IPA allows more precise DOI measurements than standard methods. The authors conducted experiments to validate the DOI Scanner’s performance and compared the results with those obtained using other DOI measurement methods. The results of the experiments show that the DOI scanner provided accurate and reliable measurements of DOI and that it was well-suited for measuring DOI on high-gloss surfaces. The protocol of the psychophysical experiment is poorly described.

Table 4 provides a brief overview of the reviews in this section of the research on distinctness-of-image gloss measurement. All three papers focus on measuring the quality of high-gloss surfaces, but they approach the problem from slightly different perspectives and use different methods. It is shown that for the measurement of DOI, there are different techniques, like a combination of light projection and imagining, to obtain the distinctness of the image, or light projection onto the sample. Furthermore, all agree that the DOI is an important parameter for quality control, especially in industries that use steel, e.g., the automotive industry. In search of papers that are related to the distinctness-of-image gloss, the work conducted in this field mostly focuses on the objective evaluation of the distinctness of image (or image clarity). Visual assessment of distinctness-of-image gloss is rarely used.

### 4.3. Gloss and Color

**Figure 4:**
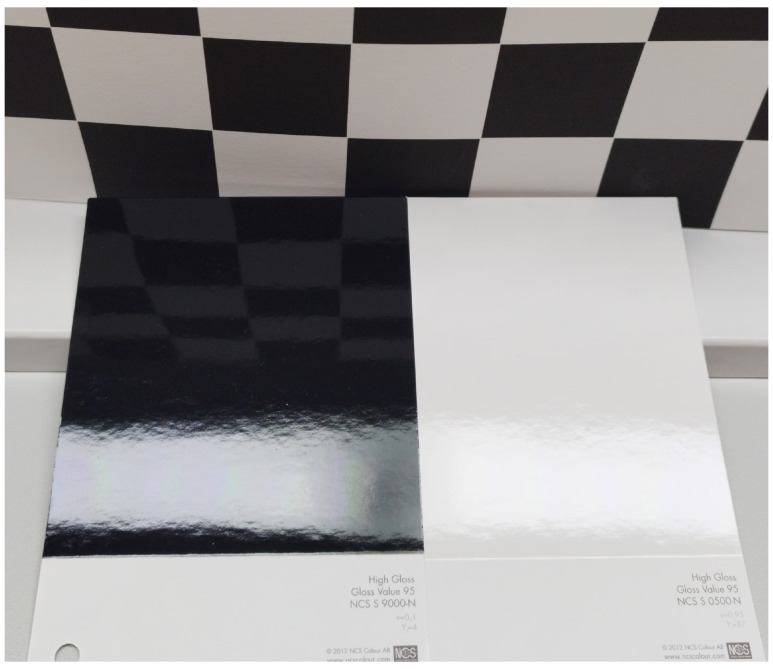
Samples with the same specular gloss but different color.

Color and gloss are two key attributes that contribute to the visual appearance of surfaces. They are distinct properties, but they are often closely related, as changes in one can affect the perception of the other. The interaction between light and surface can cause changes in both gloss and color, depending on a range of factors, such as the composition and structure of the material, the angle of incidence and reflection of the light, and the viewing conditions. For example, a smooth and uniform surface with a high gloss value will reflect light more uniformly and create a clearer, more vibrant color appearance. In contrast, a rough or irregular surface with a low gloss value may scatter and diffuse light more, creating a duller, less saturated color appearance. The relationship between color and gloss is complex and multifaceted and can be influenced by a range of factors. An example of two samples with the same specular gloss and different color is shown in Figure 4. By understanding and controlling both attributes, manufacturers and designers can create surfaces that meet the desired aesthetic and functional requirements for a range of applications.

A lot of research has been carried out in the field of interaction between gloss and color, since these are the two most important appearance attributes [45,55,78,79,80]. The contrast gloss described by Hunter and Harold in 1987 seems to indeed be a crucial dimension in gloss perception [6]. Contrast gloss is more of a subjective perception rather than a standardized measurement. It refers to the perceived difference in glossiness between two adjacent areas on a surface, which can be influenced by various factors such as surface texture, reflectance, and lighting conditions [81,82]. Because of contrast gloss, darker objects are perceived as glossier, and glossier objects are perceived as darker.

Wendt et al., in 2010, showed that by including the color information in the object, the gloss constancy performance can be improved (although gloss was classified using only specular highlights) [83]. The availability of color information has led to a significant improvement in consistency in glossiness matching compared with grayscale surfaces. Further, when observing the surface appearance, observers use different cues separately and in combination when assessing appearance. The cues can be highlight disparity, motion, and color.

In 1988, Klinker et al. demonstrated that the reflected light from every point on a dielectric object can be described as a linear combination of the object color and the highlight color (specular reflection) [84]. Their work fostered further research and exploration, particularly in the computer science domain. For instance, subsequent studies such as those from Van De Weijer and Maxwell have extended and refined the concepts introduced by Klinker et al., offering valuable insights into the dichromatic reflection model and its potential applications [85,86]. Their method separates the color from the highlight based on the chromaticity of each pixel. The authors validate their method by applying it to a set of test images and comparing the results to subjective evaluations of the highlights by human observers. They show that their method can identify highlights that are consistent with human perception, and they demonstrate the usefulness of their method for a variety of applications, including image compression, image editing, and image retrieval. Later, Nishida and Shinya studied the gloss and lightness variations in 3D objects [40]. In their experiments, observers were asked to match two 3D objects by changing the diffuse and specular reflectance. The results show that there were systematic biases in the matches as the object’s shape varied between the test and the match. They were able to model the matches on the assumption that a perceptual match occurred when the luminance histograms of the images of the test and match were similar. The results of the experiment suggest that humans use both luminance and color information to make judgments about surface reflectance properties. In particular, the authors find that luminance is an important cue for judging glossiness, while color is important for judging transparency and translucency. The authors also find that different types of color information (e.g., chromaticity, saturation) are more or less important for different types of surface properties. Furthermore, their experiment showed that the observers had trouble matching surface properties when the test and match objects had different shapes. Motoyoshi et al. asked observers to rate the lightness and glossiness of images of grayscale stucco-like materials whose space-averaged luminance was held fixed [87]. For these stimuli, they found a considerable decrease in lightness evaluation as the glossiness of the materials increased. The authors also propose a computational model that captures some of the statistical relationships between image properties and surface qualities observed in their experiments. The model is based on the idea that surface properties can be estimated by analyzing the statistical properties of the luminance and color distributions in natural images. These results are consistent with the ones from Xiado et al. [88]. In particular, they found that when the mean luminance was held fixed, the skewness of the histogram was negatively correlated with perceived lightness and positively correlated with perceived glossiness.

In Table 5, a short summary of the papers reviewed in this section is shown. The papers reviewed in this section focus on the influence of color on gloss perception. Most of the work was conducted by creating virtual stimuli, and in the papers, different materials were recreated. They all use different methodologies and approaches to study this topic, but the results are unanimous: there is an influence of color on gloss perception, in particular, the lightness (L*) part of the color. This has already been proven by previous work carried out in the field of appearance. These papers all contribute to our understanding of how humans perceive and judge surface properties in images and highlight the complex interplay between different visual cues in this process. They use a variety of methods, including psychophysical experiments, image analysis, and computer simulations, to explore different aspects of the problem. While each paper contributes unique findings, they all demonstrate the importance of considering both the physical properties of the surface and the statistical properties of the image in understanding how we perceive surface qualities.

### 4.4. Gloss and Texture

Texture and gloss are appearance attributes that contribute to the visual appearance and tactile feel of surfaces. While they are distinct properties, they are often closely related, as changes in one can affect the perception of the other. Texture refers to the surface characteristics of a material, such as its roughness, smoothness, or pattern. The interaction between surface texture and light can cause changes in the perceived gloss, depending on a range of factors such as the angle of incidence and reflection of the light, the roughness of the surface, and the spatial frequency of the surface texture. In general, a smooth and uniform surface will have a higher gloss value than a rough or irregular surface, as the smooth surface will reflect light more uniformly and create a clearer, mirror-like reflection. However, the relationship between texture and gloss is more complex. This is because the spatial frequency of surface texture, or the scale of its features, can also play an important role in determining the perceived gloss of a surface.

Numerous studies suggest that the visual system uses the correlation between surface reflectance and texture roughness properties as a cue to distinguish subjective material glossiness. Trujillo Vasquez et al. created 2.5D printed samples with different levels of surface roughness [89]. The roughness of the samples was modulated with the Perlin function setting. Gloss measurements show that there is indeed a correlation between roughness and gloss. The minimum gloss is obtained for a higher amplitude and a higher number of octaves, persistence, and amplitude. However, the study is limited in that it only investigates the influence of procedural noise on a specific printing technique and only uses a single measure of glossiness perception. Baar et al. [90] investigated the interrelation of gloss and texture with 2.5D printed samples. In the study, they conducted two psychophysical experiments: the influence of texture on gloss and the influence of gloss on texture. Results from the first experiment show that there is a slight influence of texture on the perceived glossiness. Glossy textured samples are perceived to be glossier than flat ones. On the other hand, mat samples without applied varnish are perceived as more mat with an increase in texture level. Contrarily, results obtained from the second experiment do not show an influence of gloss on the perceived texture. However, the study was limited to a relatively small set of samples.

Qi et al. [91] used a measure representing the “highlight strength” of their computer-generated stimuli, which was defined as the mean intensity of the highlights. The authors propose a model to explain why rough surfaces can appear glossy even though they scatter light in many directions, which usually results in a mat appearance. They found a significant correlation of *p* = 0.77 between the highlight strength and the glossiness judgments of their subjects. The authors use this model to predict the perceived glossiness of a set of simulated surfaces with varying roughness and specular reflection properties. Unfortunately, in the paper, the exact question that was asked to the observers in experiment 2 is not described; therefore, it is a little bit harder to understand the procedure. In Table 6, the papers mentioned in this section are summarized.

### 4.5. Gloss and Translucency

**Figure 5:**
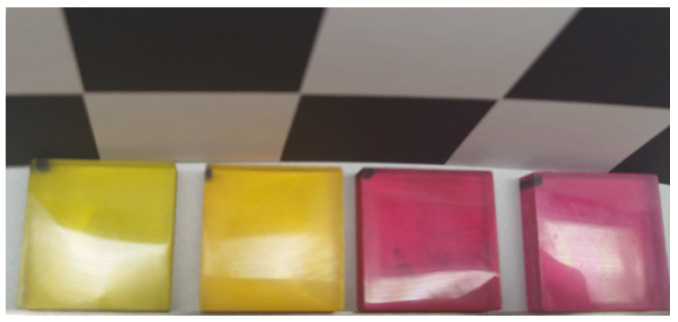
Yellow and magenta 3D printed samples with different translucency.

In the previous sections, all the samples of the work conducted in the field of gloss and gloss perception research were opaque. Therefore, in this section, the focus is on work that has been carried out in the field of gloss research with translucent stimuli. Translucency, according to Hunter [6], is the property of a material in which a large part of the transmitted light scatters. Translucency, much like gloss, plays a crucial role in the perception of an object’s appearance. However, what sets translucency research apart is the fundamental distinction in the samples being studied. This key difference is what distinguishes translucency perception, as it involves investigating light scattering rather than reflecting from a surface [92]. An example of translucent samples is shown in Figure 5. Consequently, studying the combined perception of translucency and gloss requires a comprehensive analysis of how these materials interact with light, making it a multifaceted and intricate research domain.

In 2010, Motoyoshi investigated how the highlight–shading relationship affects the perception of translucent and transparent materials. The author conducts two psychophysical experiments using computer-generated stimuli for complex 3D objects. The results of the experiment show that the highlight–shading relationship is an important cue for the perception of translucent materials. Specifically, the author notes that the perceived depth of the material increases as the highlight moves closer to the shaded area on the surface. This effect is more pronounced for materials with higher levels of translucency and transparency [93]. The experiments are well-designed, and the results are clearly presented and supported by statistical analyses. However, one limitation of the study is that the stimuli used were limited to computer-generated images of spheres, which may not fully capture the complexity and variety of real-world translucent and transparent materials. Nonetheless, the paper contributes to our understanding of the visual cues used for perceiving material properties and has practical implications for various fields, such as product design and computer graphics.

Kiyokawa et al. investigated how the perception of translucency is affected by the glossiness of a surface. The authors hypothesize that the perception of translucency is influenced by surface gloss, which affects the amount and distribution of light reflected from the surface. To test this hypothesis, the authors conducted two psychophysical experiments using computer-generated stimuli of spheres with varying levels of translucency and glossiness. Participants were asked to rate the degree of translucency of each image. The results of the experiments show that the perception of translucency is significantly influenced by the glossiness of the surface. Specifically, the perceived translucency increases as the glossiness of the surface increases, even when the actual level of translucency is held constant [94]. They propose a computational model based on measurable image features informative of shading relative to specular highlights. Furthermore, the discrepancy in orientation anisotropy between specular highlights and shading gradients is a beneficial cue in translucency perception, and in the perception of translucency in glossy objects, our visual system utilizes the low-level image features corresponding to the 3D shape, such as the anisotropy of luminance orientation.

Gigilashvili et al. investigated how translucency impacts gloss perception [95]. They designed different stimuli by changing the gloss and the subsurface scattering properties (translucency). The translucency was changed by varying the index of refraction (IOR), and the gloss was controlled by varying the surface roughness (alpha). The psychophysical experiment was not only performed with simple objects, like simple spheres; instead, the authors used more complex objects. It has already been proven in some previous work that the shape of an object influences the perceived gloss [96]. The observers in the experiment were asked to “click on the image that contains the glossier object”. The results show that spheres with smooth surfaces and higher translucency are considered as high gloss, followed by the dark opaque spheres for most of the observers. For complex shapes, the glossiest objects were the ones where the stimuli generated the most highlights. This work is a follow-up of another work from the same authors, where the authors investigated the behaviors of the observers when observing gloss and translucency [97]. The other part of the work assesses the impact of shape on translucency, but it also mentions the impact of gloss and albedo. Since the gloss is varied with the alpha parameter, which mostly influences roughness, this part of the work is considered out of the scope of this literature review paper.

These three papers (Table 7) all focus on the perception of material properties related to translucency and glossiness, but they take different approaches. They use different experimental methods to manipulate object properties and measure participant responses. Additionally, they all provide insights into the factors that influence object appearance; subsurface scattering plays a significant role in the perception of gloss, and a lack of subsurface scattering can make objects appear mat. Nevertheless, gloss and gloss highlights can provide a strong cue for the perception of translucency. Overall, these three papers provide valuable insights into the complex interplay between various cues for perceiving material properties related to translucency and glossiness. All three works are based on psychophysical experiments carried out with computer-generated stimuli. The results from both works point out that there is indeed an influence of gloss on the translucency perception among other attributes, such as, for example, the shape and the albedo color of the object. The approaches in the experiment are slightly different, but they all agree that there is indeed an influence of translucency on gloss perception and that there is an influence of gloss on translucency perception (even stronger than color on translucency). The differences are in the evaluation of the data and the virtual stimuli (different 3D objects and bumpy surfaces).

## 5. Summary

Gloss is an important appearance attribute. The development of 2.5D and 3D printing technology has opened up the possibility and need to control gloss output in a more precise way for gloss reproduction. There is a higher need to control the gloss output. In this review article, the following was carried out:

- Some of the main problems in gloss and gloss perception are discussed, as well as the work that has been carried out till now to address them. However, further research and standardization seems necessary to better manage gloss and gloss perception.
- We address attribute gloss considering material optical properties, psychophysical setups, and the questions asked during psychometric experiments.
- We systematically examine the research on gloss and gloss perception, offering a comprehensive overview of key findings and insights, as well as differences between different psychophysical experimental setups. This provides a deeper understanding of this critical aspect of surface appearance by exploring the dimensions and parameters that shape our perception of gloss. A detailed tabulation of the reviewed literature, encompassing experimental methodologies, is available in the Appendix A.

Additionally, we identify possibilities for future research, aiming to further advance the field’s understanding of gloss and its application in industrial contexts.

## Figures and Tables

**Table 1:** Short summary of the papers reviewed in the Section 2.1.2.

Author	Obein et al. [12]	Ged at al. [45]	van Assen et al. [48]			Faul et al. [51]			Mizushima S. [52]
Title	Difference scaling of gloss: Nonlinearity, binocularity, and constancy	Assessing gloss under diffuse and specular lighting	Highlight shapes and perception of gloss for real and photographed objects			Influence of Fresnel effects on gloss perception			Diffuseness of illumination suitable for reproducing a faithful and ideal appearance of an object
Open-access	YES	YES	YES			YES			YES
Samples	Real-world samples	Real-world samples	Virtual stimuli	Virtual stimuli	Real-world samples	Virtual stimuli	Virtual stimuli	Virtual stimuli	Real-world samples
Materials	Black coated paper	Paper-like	Simulated	Simulated	Glass	Simulated	Simulated	Simulated	4 different materials
Number of participants	6	29 (14M/15F)	11 (4M/7F)	11 (8M/3F)	8 (5M/3F)	3	6	5	5 (3M/2F)
Scaling method	Pair-to-pair comparison	Pair-to-pair comparison	Pair comparison	Rating task	Comparison task	Matching task	Pair comparison	Rating task	Judgment task
Question to the observers	Which pair exhibits the larger difference?	Which of these two pairs exhibits a higher difference?	Chose the glossier sphere of the two	Rate the gloss of the object in the viewing by placing a dot on the rating bar		Match the perceived gloss of the 1. and 2. object	Judge the similarity of gloss and brightness distribution on a scale 0–5	Judge gloss impression in the test object with respect to gloss level quality	Not stated
Reference scale?	NN ^1^	NN	NN	NN	Just min and max	NN	NN	NN	NN
Samples still available?	YES	YES	YES	YES	YES	NO	NO	NO	YES

^1^ NN—Not needed.

**Table 2:** Short summary of the papers reviewed in the Section 3.

Author	Ged et al. [56]	Ferwerda at al. [55]		Leloup et al. [57]	Toscani et al. [58]		Löw et al. [59]
Title	Recognizing real materials from their glossy appearance	A psychophysically based model for surface gloss perception		Overall gloss evaluation in the presence of multiple cues to surface glossiness	Three Perceptual dimensions for specular and diffuse reflection		BRDF Models for accurate and efficient rendering of glossy surfaces
Open-access	YES	NO		NO	YES		YES
Samples	Real-world samples	Virtual stimuli	Virtual stimuli	Virtual stimuli	Virtual stimuli	Virtual stimuli	Virtual stimuli
Materials	Plexiglas (PMMA)	Simulated	Simulated	Glass and paper-like samples simulated	Simulated	Simulated	Simulated
Number of participants	33	9	9	15 (7F/8M)	8	8	X
Scaling method	Pair comparison	Pair comparison	Magnitude estimation	Pair comparison	Best match task	Rating task	X
Question to the observers	Select a sample with higher perceived gloss	Judge the apparent difference in gloss between the two samples	Judge the apparent glossiness of the object in the image on a scale from 0 to 100 by adjusting the slider	Rate the glossiness of the left stimulus “i” as compared with the right stimulus “j”, using the following preference scale: (2) is much more glossy than j, (1) is more glossy than j, (0) equal, (−1) less glossy than j, (−2) is much less glossy than j	Which of the 4 objects is most similar to the object on the right in terms of surface material properties	Indicate how much each adjective is appropriate to describe the surface of the shape presented on screen, from 0% to 100%	X
Samples still available?	YES	YES	YES	YES	YES	YES	YES
Dimensions	3 (Haze, DOI and Roughness type)	2 (DOI and contrast gloss)		DOI and L* one of the cues	3 (Gloss, L* and metalicity)		3 (Attenuation, Beckmann distribution, and clarity)

**Table 3:** Short summary of papers reviewed in the Section 4.1.

Author	Vangorp et al. [67]			González-Leal et al. [66]
Title	Perception of Hazy Gloss			A novel method for assessing haze in the visual appearance of bright-annealed AISI 430 ferritic stainless steel
Open-access	YES			YES
Samples	Virtual stimuli	Virtual stimuli	Virtual stimuli	AISI 430 ferritic stainless steel
Materials	Simulated	Simulated	Simulated	Real-world samples
Number of participants	NA	9	14	0
Scaling method	Matching task	Discrimination task	Rating task	NN
Questions in the experiment	Adjust a single parameter of the object on the right until it appears to be the same material as the object on the left	Which material looks different from the others in terms of the sharpness or blurriness of the reflection?	Rate the presented material on the following six different continuous scales related to gloss appearance	NN
Reference scale	NN	NN	Just min and max	NN
Samples still available?	YES	YES	YES	YES

**Table 4:** Short summary of papers reviewed in the Section 4.2.

Author	Tse and Biggs [76]	Lu, Ren, Wen and Li [75]	Gruber and Buder-Stroisznigg [77]
Title	A new measurements device for measurement of DOI	Relationship between distinctness of image of organic coatings and texture substrate sheets	Measuring distinctness of image on high-gloss surfaces
Open-access	YES	YES	YES
Samples	Real-world samples	Real-world samples	Real-world samples
Materials	Prints on paper	Steel sheet	Coated steel
Number of participants	NA	NA	25
Scaling method	No psychophysical experiment	No psychophysical experiment	Ranking task
Questions in the experiment	/	/	Not stated
Reference scale	NN	NN	NO
Samples still available?	YES	YES	YES

**Table 5:** Short summary of papers reviewed in the Section 4.3.

Author	Klinker et al. [84]	Wendt et al. [83]	Xiao and Brainard [88]		Nishida and Shinya [40]	Motoyoshi et al. [87]	
Title	Measurement of highlights in color images	Disparity, motion, and color information improve gloss constancy performance	Surface gloss and color perception of 3D objects		Use of image-based information in judgments of surface reflectance properties	Image statistics and the perception of surface qualities	
Open-access	YES	YES	NO		NO	NO	
Samples	Digital images	Virtual stimuli	Virtual stimuli		Virtual stimuli	Virtual stimuli	
Materials	Plastic, paper, ceramic	Simulated	Simulated		Simulated	Stucco-like	
Number of participants	/	4	7		5	6	
Scaling method	Image analysis	Matching task	Matching task	Matching task	Matching task	Unclear	Pair comparison
Questions in the experiment	/	Match the perceived lightness and glossiness closely as possible by adjusting the values of the diffuse component and the Phong exponent	Match the color appearance of the mat sphere to that of the test sphere	Match the color appearance of the two mat spheres	Change the reflectance parameters of the surface to match the two surfaces	Not stated	Not clear

**Table 6:** Short summary of the papers reviewed in the Section 4.4.

Author	Trujillo Vasquez et al. [89]	Baar at al. [90]		Qi et al. [91]	
Title	Influence of procedural noise on the glossiness of 2.5D printed samples	Interrelation between gloss and texture perception of 2.5D printed surfaces		Why do rough surfaces appear glossy?	
Open-access	YES	YES		NO	
Samples	Real-world samples	Real-world samples	Real-world samples	Virtual stimuli	Virtual stimuli
Materials	2.5D prints	2.5D prints	2.5D prints	Simulated	Simulated
Number of participants	/	15 (6F/9M)	15 (6F/9M)	5	9
Scaling method	/	Rating task	Rating task	Rating task	Not clear
Questions to the observers	/	Select the reference sample with the same perceived gloss as the test sample	Assign texture scale value of the ref. samples that match the texture of the test sample	Is the surface mat or glossy?	Unclear
Reference scale	/	NCS Gloss scale	Authors own prints	NN	X
Samples still available?	YES	YES	YES	YES	YES

**Table 7:** Short summary of the papers reviewed in the Section 4.5.

Author	Gigilashvili et al. [95]	Kiyokawa et al. [94]		Motoyoshi et al. [93]	
Title	The role of subsurface scattering in glossiness perception	The perception of translucency from surface gloss		Highlight–shading relationship as a cue for the perception of translucent and transparent materials	
Open-access	YES	NO		YES	
Samples	Virtual stimuli	Virtual stimuli		Virtual stimuli	
Materials	Simulated	Simulated		Simulated	
Number of participants	250	18 (15M/3F)	6 (4M/2F)	8 (5F/3M)	7
Scaling method	Pair comparison	Pair comparison	Rating task	Rating task	Rating task
Questions to the observers	Click on the image that contains the glossier object	Which of the two images appears more translucent?	Evaluate the bumpiness of the 3D sample in a range from 0–4	Rate the object’s apparent translucency on a five-point scale: opaque (0), translucent like marble wax (1–2), highly translucent like jelly or glass (3–4)	
Samples still available?	YES	YES	YES	YES	YES

## Data Availability

Not applicable.

## Appendix A

**Author**	**Title**	**Open-Access**	**Samples**	**Materials**	**Number of Participants**	**Task**	**Questions in the Experiment**	**Samples Still Available**	**Reference Scale?**	**Dimensions**
Obein et al. [12]	Difference scaling of gloss: nonlinearity, binocularity, and constancy	YES	Real-world samples	Black-coated paper	6	Pair-to-pair comparison	“Which pair exhibits larger difference?”	YES	NN	
Ged at al. [45]	Assessing gloss under diffuse and specular lightning	YES	Real-world samples	Paper-like	29 (14M/15F)	Pair-to-pair comparison	“Which of the two pairs exhibits a higher difference?”	YES	NN	
van Assen et al. [48]	Highlight shapes and perception of gloss for real and photographed objects		Virtual stimuli	Simulated	11 (4M/7F)	Pair comparison	“Chose the glossier sphere from the two?”	YES	NN	
			YES	Virtual stimuli	Simulated	11 (8M/3F)	Rating task	“Rate the gloss of the object in the viewing booth by placing dot on the rating bar”	YES	NN	
				Real-world samples	Glass	8 (5M/3F)	Comparison task	"Rate the gloss of the object in the viewing booth by placing dot on the rating bar"	YES	Just min and max	
Faul at al. [51]	Influence of Fresnel effects on gloss perception		Virtual stimuli	Simulated	3	Matching task	"Match the perceived gloss of the first and second object"	NO	NN	
			YES	Virtual stimuli	Simulated	6	Pair comparison	“Judge the similarity of gloss and brightness distribution on a scale from 0 to 5”	YES	N0	
				Virtual stimuli	Simulated	5	Rating task	“Judge gloss impression in the test object with respect to gloss level quality”	YES	NO	
Mizhushima S. [52]	Diffuseness of illumination suitable for reproducing a faithful and ideal appearance of an object	YES	Real-world samples	Paper-like	29 (14M/15F)	Pair-to-pair comparison	"Which of the two pairs exhibits a higher difference?"	YES	NN	
Ged at al. [56]	Recognizing real materials from their glossy appearance	YES	Real-world samples	Plexiglas (PMMA)	33	Pair-to-pair comparison	"Select sample with higher perceived gloss!"	YES	NN	3
Ferwerda et al. [55]	A psychophysically based model for surface gloss perception	NO	Virtual stimuli	Simulated	9	Pair comparison	"Judge the apparent difference in gloss between the two samples"	YES	NN	2
			NO	Virtual stimuli	Simulated	9	Magnitude estimation	"Judge the apparent glossiness of the object in the image from 0 to 100 by adjusting the slider"	YES	NO	2
Leloup et al. [57]	Overall gloss evaluation in the presence of multiple cues to surface glossiness	NO	Virtual stimuli	Glass and paper-like	8	Pair comparison	"Rate the gloss of the left stimulus "i" as compared with the stimulus "j" using the scale"	YES	NN	3
Toscani et al. [58]	Three perceptual dimensions for specular and diffuse reflection	YES	Virtual stimuli	Simulated	8	Best match task	"Which of the 4 objects is most similar to the object on the right in terms of surface material properties?"	YES	NN	3
				Virtual stimuli	Simulated	8	Rating task	Indicate how much each adjective is appropriate to describe the surface of the shape presented, from 0% to 100%"	YES	NO	3
Löw et al. [59]	BRDF Models for accurate and efficient rendering of glossy surfaces	YES	Virtual stimuli	Simulated	X	X	X	YES	NN	3
Vangorp et al. [67]	Perception of Hazy gloss		Virtual stimuli	Simulated	NA	Matching task	“Adjust a single parameter of the object on the right until it appears to be same material as the object on the left”	YES	NN	
			YES	Virtual stimuli	Simulated	9	Discrimination task	“Which material looks different from other in terms of sharpness or blurriness of the reflection?”	YES	NO	
				Virtual stimuli	Simulated	14	Rating task	“Rate the presented material on the 6 scales related to gloss appearance”	YES	Just min and max	
Gonzales - Leal et al. [66]	A novel method for assessing haze in the visual appearance of bright-annealed ASI 430 ferritic stainless steel	YES	Real-world samples	AISI 430 steel	0	NN	NN	YES	NN	
Tse and Biggs [76]	A New Instrument for Distinctness of Image (DOI) Measurements	YES	Real-world samples	2D prints	NA	X	X	YES	NN	
Lu, Ren, Wen and Li [75]	Relationship between DOI of organic coatings and texture substrate sheets	YES	Real-world samples	Steel sheet	NA	X	X	YES	NN	
Gruber and Bruder-Strisznigg [77]	Measuring Distinctness of Image of High Gloss surfaces	YES	Real-world samples	Coated steel	25	Ranking task	Not stated	YES	N0	
Klinker et al. [84]	The Measurement of Highlights in Color Images	YES	Digital Images	Plastic, paper, ceramics	X	Image analysis	X	YES	X	
Wendt et al. [83]	Disparity, motion, and color information improve gloss constancy performance	YES	Virtual stimuli	Simulated	4	Matching task	“Match the perceived lightness and glossiness as closely as possible by adjusting the values of the diffuse component and the Phong exponent”	YES	X	
Xiao and Brainard [88]	Surface gloss and color perception of 3D objects	NO	Virtual stimuli	Simulated	7	Matching task	“Match the color appearance of the mat sphere to that of the test sphere”		NN	
				Virtual stimuli	Simulated	7	Matching task	“Match the color appearance of the two mat spheres”	YES	NN	
Nishida and Shinya [40]	Use of image-based information in judgments of surface reflectance properties	NO	Virtual stimuli	Simulated	5	Matching task	“Change the reflectance parameters of the surface to match the two surfaces”	YES	NN	
Motoyoshi et al. [87]	Image statistics and the perception of surface qualities	NO	Virtual stimuli	Stucco-like	6	Unclear	Not stated	YES	NN	
				Virtual stimuli	Stucco-like	6	Pair comparison	Not clear	YES	NN	
Trujillo Vasquez et al. [89]	Influence of procedural noise on the glossiness of 2.5D printed patches	YES	Real-world samples	2.5D prints	X	X	X	YES	X	
Baar et al. [90]	Interrelation between gloss and texture perception of 2.5D-printed surfaces	YES	Real-world samples	2.5D prints	15 (6F/9M)	Rating task	“Select the reference sample with the same perceived gloss as the test sample”	YES	NCS Gloss scale	
				Real-world samples	2.5D prints	15 (6F/9M)	Rating task	Assign texture scale value of the ref.samples that match the texture to the test sample	YES	Authors own prints	
Qi et al. [91]	Why do rough surfaces appear glossy?	NO	Virtual stimuli	Simulated	5	Rating task	“Is the surface mat or glossy?”	YES	NN	
				Virtual stimuli	Simulated	9	Not clear	Unclear	YES	X	
Gigilashvili et al. [95]	The role of subsurface scattering in glossiness perception	YES	Virtual samples	Simulated	250	Pair comparison	"Click on the image that contains the glossier object”	YES	NN	
Kiyokawa et al. [94]	The perception of translucency from surface gloss	NO	Virtual stimuli	Simulated	18 (15M/3F)	Pair comparison	"Which of the two images appears more translucent?”	YES	NN	
				Virtual stimuli	Simulated	6 (4M/2F)	Rating task	“Evaluate the bumpiness of the 3D samples in a range from 0 to 4"	YES	NN	
Motoyoshi et al. [93]	Highlight-shading relationship as a cue for the perception of translucent and transparent materials.	YES	Virtual stimuli	Simulated	8 (5M/3F)	Rating task	“Rate the object’s translucency on a five-point scale”	YES	NO	
				Virtual stimuli	Simulated	7	Rating task	“Evaluate the bumpiness of the 3D samples in a range from 0 to 4”	YES	NN	
